# Assessment of Liver Transplant Eligibility in Chronic Liver Disease Patients: A Cross-Sectional Study From a Tertiary Care Hospital of Pakistan

**DOI:** 10.7759/cureus.61028

**Published:** 2024-05-24

**Authors:** Subtain Hassan, Suleman Khan, Asif Khan, Mahnoor Khattak, Ehtisham K Khattak, Ameer M Farrukh, Qaisar Ali Khan

**Affiliations:** 1 Medicine, MTI Khyber Teaching Hospital (KTH), Peshawar, PAK; 2 Internal Medicine, Rehman Medical Institute, Peshawar, PAK; 3 Medicine, Khyber Medical University (KMU) Institute of Medical Sciences Kohat, Kohat, PAK; 4 Medicine, University of Galway, Galway, IRL; 5 Internal Medicine, MTI Khyber Teaching Hospital (KTH), Peshawar, PAK; 6 Internal Medicine, DHQ Teaching Hospital Kohat, Kohat, PAK

**Keywords:** chronic liver diseases, end-stage liver disease, liver transplant, meld score, liver cirrhosis

## Abstract

Introduction: The burden of chronic liver disease (CLD) is increasing globally and the ultimate treatment is a liver transplant. As Pakistan is a developing country, liver transplantation is not easily available due to limited resources. This study aims to assess the patients with CLD for liver transplantation and to find the frequency of eligible candidates for liver transplantation.

Methods: A cross-sectional observational study was conducted on patients with CLD from June 2022 to December 2022. Total bilirubin, serum creatinine complete blood count, serum electrolytes, and international normalised ratio (INR) were done. The Model for End-Stage Liver Disease (MELD) score was calculated and the frequency of eligible patients for liver transplant was determined. Data was entered and analyzed using Statistical Package for Social Sciences (SPSS) version 22 (IBM Corp., Armonk, NY, USA).

Results: In our study, 149 patients were enrolled with a mean age of 46.81±15.7 years. There were 58.7% male and 41.6% female patients. The mean duration of liver cirrhosis was 18.22±11.7 months. The mean MELD score was 20.71±5.2. The common liver cirrhosis stages were stage II and stage II was found in 32.2% of each. Hepatocellular carcinoma (HCC) was present in 15.4% of patients. There were 25.5% of patients eligible for liver transplants.

Conclusion: In our study, we found that significant numbers of patients with CLD were eligible for liver transplantation.

## Introduction

Chronic liver disease (CLD) is the 12th leading cause of mortality in the United States. Hepatocellular carcinoma (HCC) accounts for the majority of CLD cases in the United States [1]. Pakistan ranks moderate for hepatitis B and C, which cause more than 75% of liver cirrhosis and hepatocellular cancer in the WHO Eastern Mediterranean Regional Office (EMRO) region [2]. Non-alcoholic fatty liver disease (NAFLD) increases the frequency of cirrhosis and HCCs requiring liver transplantation (LT) [3]. Before liver transplantation, acute liver failure and cirrhosis were treated by alleviating symptoms. Effective liver transplantation improves life quality and lifespan [4]. In the previous few years, more than 5000 liver transplants have been performed in Europe, bringing the total to more than 140,000, resulting in a good post-LT survival rate of 80% in one year and 90% in five years [4,5]. Indications for liver transplant in adults include acute liver failure, cirrhosis from chronic liver diseases, metabolic disorders originating from the liver, liver-related malignancies, polycystic liver disease, hereditary hemorrhagic telangiectasia, and erythropoietic porphyria. Cirrhosis from chronic liver disease is the most common reason for liver transplantation [6]. 

The transplant evaluation technique focuses on operational risk assessment, medical compliance, and co-morbidities that could affect graft survival and overall survival with immunosuppressive drugs [7]. Hepatic cirrhosis and its systemic effects almost always need transplantation in chronic liver disease patients [8]. Screening patients for liver transplant eligibility is the most important stage. Thus, managing waiting list patients is crucial to preventing mortality and sliding off the list, and boosting post-transplant survival [9]. Intermediate Model for End-Stage Liver Disease (MELD) scores and liver cirrhosis patients can wait over a year on the waiting list for transplantation [10]. Effective selection of patients for liver transplantation in chronic liver illnesses lowered ICU stays to less than 10% [4]. Vugts et al. concluded in their study seeking eligibility for liver transplantation that among a total of 732 patients with chronic liver failure, only 5% were eligible for liver transplantation [10]. Chronic liver diseases are responsible for a large portion of the country’s economic burden. Among Asian countries, Pakistan has the greatest frequency of chronic liver disease. The majority of the patients present with complications due to poor compliance with medications, a lack of satisfactory counseling, late presentation at the time of diagnosis, overuse of Hakimi medications, and a lack of expert centers to deal with these cases across the country. In Pakistan, the need for liver transplantation has risen in recent years. As a result, the first step toward liver transplantation and post-transplant surveillance is proper screening for eligible candidates. The study aims to assess the patients with CLD for liver transplantation and to know the frequency of eligible patients. 

## Materials and methods

Study design and sample size

A cross-sectional study was conducted at the Department of Medicine, Khyber Teaching Hospital, Peshawar, spanning from June 27, 2022, to December 26, 2022. The sample size was determined to be 149 chronic liver disease patients based on a 5% prevalence of eligible candidates for liver transplantation in patients with chronic liver failure. This calculation was done using a 95% confidence interval and a 3.5% margin of error, as per the WHO calculator for sample size determination, where a smaller margin of error necessitates a larger sample size.

Sampling technique

The sampling technique employed was non-probability consecutive sampling, whereby participants were selected based on their availability and willingness to participate.

Inclusion criteria

Inclusion criteria include patients diagnosed with chronic liver disease secondary to chronic hepatitis B and C, Wilson disease, autoimmune hepatitis, and hepatocellular carcinoma, regardless of gender, within the age range of 18 to 70 years.

Exclusion criteria

Exclusion criteria were established to refine the study population and exclude confounding factors. These included patients with malignancies outside the liver, a history of alcoholism spanning at least six months, substance abuse, active infections, disabling psychiatric diseases, poor compliance with medications, prior radiotherapy to the abdomen, lack of family support, and unwillingness to provide informed consent.

Data collection

Khyber Medical College Peshawar Institutional Research and Ethical Review Board (IREB) issued approval 304/DME/KMC. All patients with chronic liver disease who met the inclusion criteria were evaluated after receiving written consent from College of Physicians and Surgeons Pakistan (CPSP). These patients were requested to sign an informed consent form in writing. A detailed medical history was taken from these patients, followed by a physical examination. All willing chronic liver disease patients were evaluated for liver transplantation eligibility. A phlebotomist was called up, and blood was taken for all relevant tests, including biochemical tests like total bilirubin, serum creatinine complete blood count, serum electrolytes, and international normalised ratio (INR). The etiology of the chronic liver disease was identified by serological tests or polymerase chain reaction (PCR). The metabolic screening was done for hemochromatosis and Wilson disease. HCC was diagnosed and staged based on a biopsy and CT scan. These patients were tested for anesthesia fitness as well. On a pre-designed proforma, all demographic information, patients' laboratory investigations, scans, and the findings of numerous tests were recorded. To eliminate any confounding factor or bias from the study, strict exclusion criteria were used.

Data analysis

Statistical Package for Social Sciences (SPSS) version 22.0 (IBM Corp., Armonk, NY, USA) was used for the analysis of data. The mean and standard deviation were computed for categorical variables like age, disease duration, and MELD scores. Frequency and percentages were calculated for categorical variables such as gender, stage of liver cirrhosis, presence of HCC, and frequency of eligible candidates. The frequency of eligible candidates was stratified based on age, gender, duration of the disease, etiology of the chronic liver disease, and presence of HCC to see the effect modifications. Post-stratification, the chi-square test was applied to keep the p-value ≤0.05 as statistically significant.

## Results

A total of 149 patients were assessed in this study. The mean age of the participants was 46.81±15.7 years. There were 58.4% (87) male and 41.6% (62) female patients. Regarding the stages of liver cirrhosis, 14 (9.4%) have Stage I, 48 (32.2%) have Stage II and Stage III and 39 (26.2%) have Stage IV liver cirrhosis. Twenty-three (15.4%) patients have concurrent HCC. The mean duration of liver cirrhosis was 18.22±11.7 months. The summary of participants’ demographic information is given in Table 1.

**Table 1:** Participants' demographic information HCC: Hepatocellular Carcinoma

Variable	Number (n)	Percentage
Gender		
Male	87	58.4%
Female	62	41.6%
Stages of Liver Cirrhosis		
Stage I	14	9.4%
Stage II	48	32.2%
Stage III	48	32.2%
Stage IV	39	26.2%
Presence of HCC		
Yes	23	15.4%
NO	126	84.6%
Cause of Liver Cirrhosis		
Hepatitis B	36	24.1%
Hepatitis C	107	71.8%
Autoimmune Hepatitis	4	2.7%
Wilsons Disease	2	1.3%

The mean MELD score of the patient was 20.71, and 38 (25.5%) patients were eligible for liver transplantation. The results were further analyzed based on age, gender, duration of cirrhosis, etiology, and presence of HCC. No statistically significant difference was noted in the eligibility of liver transplantation based on age groups, duration of disease, etiology of the disease and presence of HCC was noted except in terms of gender. Female patients were more eligible for liver transplantation than males with a p-value of 0.001. The elaborated results are given in Table 2.

**Table 2:** Stratified analysis of liver transplant-eligible patients HCC: Hepatocellular carcinoma

Variables			Eligible for transplant		Total	P value
			Yes	No		
Age groups	18-45 years	Count	12	54	66	0.067
		% within Age groups	18.2%	81.8%	100.0%	
	46-70 years	Count	26	57	83	
		% within Age groups	31.3%	68.7%	100.0%	
Gender	Male	Count	10	77	87	0.001
		% within Gender	11.5%	88.5%	100.0%	
	Female	Count	28	34	62	
		% within Gender	45.2%	54.8%	100.0%	
Duration of disease	Equal to or less than 18 months	Count	20	71	91	0.216
		% within Duration of disease	22.0%	78.0%	100.0%	
	More than 18 months	Count	18	40	58	
		% within Duration of disease	31.0%	69.0%	100.0%	
Etiology of chronic liver disease	Hepatitis B	Count	9	27	36	0.571
		% within Etiology of chronic liver disease	25.0%	75.0%	100.0%	
	Hepatitis C	Count	26	81	107	
		% within Etiology of chronic liver disease	24.3%	75.7%	100.0%	
	Autoimmune Hepatitis	Count	2	2	4	
		% within Etiology of chronic liver disease	50.0%	50.0%	100.0%	
	Wilson disease	Count	1	1	2	
		% within Etiology of chronic liver disease	50.0%	50.0%	100.0%	
Presence of HCC	Yes	Count	4	19	23	0.332
		% within Presence of HCC	17.4%	82.6%	100.0%	
	No	Count	34	92	126	
		% within Presence of HCC	27.0%	73.0%	100.0%	

## Discussion

Liver transplantation stands as a vital therapeutic intervention for individuals facing life-threatening liver diseases, including acute liver failure, end-stage chronic liver disease, primary hepatic cancers, and metabolic disorders. Despite an increase in deceased donor numbers, post-transplant outcomes tend to be less favorable with advanced donor age. Notably, liver transplantation has emerged as an effective treatment for HCC, contributing significantly to transplant indications. However, the persistent demand for donor organs exceeds the available supply. Globally, liver transplantation plays a pivotal role in managing end-stage liver disease, ranking second only to kidney transplantation in terms of major organ transplants. In the United States, organ allocation is governed by protocols established by the United Network for Organ Sharing (UNOS), which rely on scoring systems like MELD [11].

The demographic profile of our study population reveals insights into the age and gender distribution of patients with CLD in our setting. With a mean age of 46.81 years and a slightly higher representation of male patients (58.7%), our findings align with general trends observed in CLD populations. This distribution reflects the prevalence of liver diseases, which often exhibit a higher incidence in males and are commonly diagnosed in middle-aged individuals [12]. While our sample may not fully represent the broader CLD population, these demographic characteristics provide a foundation for understanding the epidemiology of liver diseases in our region. The distribution of liver cirrhosis stages within our study population highlights the severity and progression of liver disease among patients enrolled. The most common liver cirrhosis stage was stage II found in 32.2% each. Stage II cirrhosis emerged as the most common stage, suggesting a significant proportion of patients with moderate liver damage. This finding underscores the importance of early detection and intervention to prevent disease progression and associated complications.

The coexistence of HCC in 15.4% of patients within our study population underscores the clinical complexity inherent in managing CLD, especially when malignancy coincides with cirrhosis. The presence of HCC poses significant challenges in treatment planning and prognosis assessment, as it necessitates a multidisciplinary approach to address both the underlying liver disease and the associated cancer. The mean duration of liver cirrhosis among our study participants was 18.22±11.7 months, reflecting the chronic nature of the disease and its progressive course over time. This duration highlights the importance of early diagnosis and intervention in CLD management to mitigate disease progression and reduce the risk of complications such as decompensation, portal hypertension, and hepatocellular carcinoma development. Additionally, the relatively short mean duration of cirrhosis observed in our study population underscores the need for timely and comprehensive medical care to optimize patient outcomes and prevent disease-related morbidity and mortality.

In our study, CLD presented with diverse etiologies, yet a notable majority of patients were afflicted with hepatitis C. This observation aligns with global epidemiological trends, where hepatitis C remains a predominant cause of CLD, particularly in certain regions and demographic groups. The predominance of hepatitis C-related CLD underscores the ongoing burden of this viral infection on liver health and highlights the need for targeted prevention, screening, and treatment efforts to curb its impact. Our study findings are in line with research conducted by Syed et al., which emphasizes that hepatitis C virus remains the predominant cause of chronic liver disease necessitating liver transplantation [13].

However, the frequency of waiting list/eligible patients for liver transplant varies among different populations depending upon the stage of liver cirrhosis at the time of presentation in the hospital, MELD score, and available resources. Vugts et al. concluded in their study seeking eligibility for liver transplantation that among a total of 732 patients with chronic liver failure, only 5% were eligible for liver transplantation [10]. In another study, it was found that nonbiliary cirrhosis, particularly alcohol- and hepatitis C virus-related cirrhosis (60%), and tumors, mainly hepatocellular carcinoma (19%), are the most common indications for LT in Spain. Unusual causes of LT include metabolic diseases like Wilson's disease, familial amyloid polyneuropathy and hyperoxaluria type I, polycystic kidney and liver disease, and some tumors (epithelioid hemangioendothelioma and neuroendocrine tumors) [14]. Evaluating eligibility for liver transplantation using MELD scores indicated that 25.5% of patients fulfilled the criteria for transplantation. A study conducted in the USA revealed a noteworthy increase in liver transplant candidates among adult patients, rising from 9% between 2002 and 2005 to 23% between 2018 and 2020 (trend, p < 0.0001) [15]. This underscores a substantial rise in eligibility criteria over a decade-long timeframe.

The study's reliance on data from a single center may limit the generalizability of its findings to broader populations or regions due to potential variations in patient demographics, healthcare practices, and access to transplantation services. Additionally, a larger sample size would have strengthened the study's reliability and yielded more robust results. Recruitment from a specific healthcare facility may have introduced selection bias, as patients at this center may not fully represent the diverse population of chronic liver disease patients, especially those lacking access to specialized care. Additionally, the study's omission of certain potential confounding variables concerning transplant eligibility might result in biased estimates of association. Specifically, focusing solely on MELD scores as a determinant of transplant candidacy overlooks other relevant factors such as socioeconomic status, patient preferences, and organ availability, thus limiting the study's comprehensiveness.

## Conclusions

A significant number of patients in Pakistan meet the criteria for liver transplantation, which highlights how critical it is to address the organ shortage. As reputable community members, doctors can shape public perceptions and actions around organ donation. Furthermore, structural improvements that facilitate organ donation and transplantation processes must be implemented in concert with legislators, community stakeholders, and healthcare professionals.
